# Weight management for overweight and obese men delivered through professional football clubs: a pilot randomized trial

**DOI:** 10.1186/1479-5868-10-121

**Published:** 2013-10-30

**Authors:** Cindy M Gray, Kate Hunt, Nanette Mutrie, Annie S Anderson, Shaun Treweek, Sally Wyke

**Affiliations:** 1 Institute of Health and Wellbeing, University of Glasgow, 27 Bute Gardens, Glasgow G12 8RS, UK; 2 MRC/CSO Social and Public Health Sciences Unit, University of Glasgow, 4 Lilybank Gardens, Glasgow G12 8RZ, UK; 3 Institute of Sport, Physical Education and Health Sciences, University of Edinburgh, St Leonard's Land, Holyrood Road, Edinburgh EH8 8AQ, UK; 4 Centre for Public Health Nutrition Research, University of Dundee, Dundee DD2 4BF, UK; 5 Health Services Research Unit, University of Aberdeen, Health Sciences Building, Foresterhill, Aberdeen AB25 2ZD, UK

**Keywords:** Overweight, Obesity, Physical activity, Diet, Behaviour change, Men, Gender, Masculinities, Intervention, Sports club

## Abstract

**Background:**

The prevalence of male obesity is increasing, but men are less likely than women to attend existing weight management programmes. We have taken a novel approach to reducing perceived barriers to weight loss for men by using professional football (soccer) clubs to encourage participation in a weight management group programme, gender-sensitised in content and style of delivery. Football Fans in Training (FFIT) provides 12 weeks of weight loss, physical activity and healthy eating advice at top professional football clubs in Scotland. This pilot randomized trial explored the feasibility of using these clubs as a setting for a randomized controlled trial of 12 month weight loss following men’s participation in FFIT.

**Methods:**

A two-arm pilot trial at two Scottish Premier League football clubs (one large, one smaller), with 103 men (aged 35–65, body mass index (BMI) ≥27 kg/m^2^) individually randomized to the intervention (n=51, received the pilot programme (p-FFIT) immediately) and waitlist comparison (n=52, received p-FFIT after four months) groups. Feasibility of recruitment, randomization, data collection and retention were assessed. Objective physical measurements (weight, waist circumference, blood pressure, body composition) and questionnaires (self-reported physical activity, diet, alcohol consumption, psychological outcomes) were obtained from both groups by fieldworkers trained to standard protocols at baseline and 12 weeks, and from the intervention group at 6 and 12 months. Qualitative methods elicited men’s experiences of participation in the pilot trial.

**Results:**

Following a short recruitment period, the recruitment target was achieved at the large, but not smaller, club. Participants’ mean age was 47.1±8.4 years; mean BMI 34.5±5.0 kg/m^2^. Retention through the trial was good (>80% at 12 weeks and 6 months; >75% at 12 months), and 76% attended at least 80% of available programme delivery sessions. At 12 weeks, the intervention group lost significantly more weight than the comparison group (4.6% c.f. -0.6%, p<.001) and many maintained this to 12 months (intervention group baseline-12 month weight loss: 3.5%, p<.001). There were also improvements in self-reported physical activity and diet, many sustained long term.

**Conclusions:**

The results demonstrated the feasibility of trial procedures and the potential of FFIT to engage men in sustained weight loss and positive lifestyle change. They supported the conduct of a fully-powered randomized controlled trial.

## Background

The prevalence of male obesity is increasing worldwide [1]. In the UK, more men than women are overweight or obese (England: 65% men compared with 58% women [2]; Scotland: 69% men compared with 57% women [3]), and adult male obesity is forecast to reach 60% by 2050 [4]. Men are at increased risk of obesity-related ill health (e.g., type 2 diabetes, hypertension, dyslipidemia, cardiovascular disease, osteoarthritis and some cancers [4]), but are less likely than women to attempt to lose weight or take part in organised weight management programmes [5-9].

The apparent reluctance of men to engage in weight loss programmes may reflect the fact that overweight men tend to be less aware than women of their overweight status [10,11] and to associate increased body size with muscularity and masculinity [12,13]. Men may also harbour misperceptions about the dietary behaviours required to lose weight [11,14,15] and perceive dieting and existing organized weight loss programmes (typically female-dominated) as ‘feminized’ domains [16]. Men who want to lose weight may be more attracted to programmes that focus on physical activity as well as diet [9,17] and often express a desire to be in the company of others they feel they can identify with [11,18].

Professional football clubs are still largely a male environment, and the social and psychological connections (e.g., identity, validation, belonging) that being a fan creates are powerful [19]. There is growing recognition of the potential of professional sports organisations to attract men who are ‘hard-to-reach’ and at high risk of ill health to healthy lifestyle initiatives [20-22]. Recent evidence suggests the professional sports club setting may be effective for engaging men in sustained weight loss. For example, 40 men taking part in a men’s health initiative at Celtic and Rangers Football Clubs in Glasgow achieved an average 4% weight reduction during a 10 week programme and continued to lose weight over the following 12 months [22].

To date, evaluation of the effectiveness of delivering health promotion through professional sports clubs and men-only weight management programmes has been subject to various limitations. Studies have often focused on short-term outcomes, many have been small scale in nature, have had low response rates at follow-up, were not evaluating a ‘standardized delivery’, and none have used randomized designs [21,23-28]. Indeed others [28] have suggested that ‘hard to reach’ men may have ‘apprehensions regarding surveillance’ (p411), making it difficult to undertake data collection except through a ‘partnership model’ in which the deliverers of the intervention also collect data from participants in the evaluation [21]. This raises important questions about whether more scientifically rigorous evaluative designs, including independent and objective measurements, are possible within the professional sports club context and with this population.

Football Fans in Training (FFIT) is a gender-sensitised, weight management, physical activity and healthy eating programme developed for delivery to men through professional football clubs by community coaches trained to a standardized delivery protocol. Best practice guidance for intervention development and evaluation [29] has been followed, with iterative programme development and feasibility work being conducted prior to formal evaluation of 12 month weight loss in a randomized controlled trial (RCT). The development and optimization of FFIT for delivery through football clubs in the Scottish Premier League (SPL), the top professional league in Scotland, is described elsewhere [18]. The current paper presents the findings of the pilot randomized trial undertaken to assess the feasibility of the protocol for conducting the subsequent full RCT [30]. The aims were: 1) to evaluate the feasibility and acceptability of recruiting men to a trial of a weight management programme delivered through professional football clubs; 2) to provide an estimate of participant retention to 12 months; and 3) to explore the potential of FFIT to help men lose weight, retain that weight loss to 12 months (primary outcome in the subsequent RCT) and make positive changes to self-reported lifestyle and psychological measures (secondary outcomes in the subsequent RCT).

## Methods

### Pilot trial design

This was a two-arm, pragmatic pilot randomized trial conducted in two SPL football clubs selected to represent the diversity among clubs in the Scottish Premier League. One club was city-based with a large fan base, many of whom lived locally; the second was town-based with a smaller fan base, many of whom did not live locally. Following baseline measurement and assessment of eligibility, men in each club were individually randomized to the intervention group (starting FFIT immediately) or the waitlist comparison group (starting FFIT after a 4-month delay).

### Participants

Eligibility criteria were: male; aged 35–65 years; with a body mass index (BMI) of at least 27 kg/m^2^. These criteria were selected to maximize both potential public health gain and participant motivation to lose weight. Overweight and obese men in their mid to late 30s may experience an attitudinal shift in relation to their health and physical limitations [31], increasing their receptiveness to advice on changing health behaviours; and men who are obese (or at high risk of becoming obese) are more likely to want to lose weight than those who just exceed the normal weight range [32,33]. The upper age limit reflects differences in current physical activity guidelines for over-65s [34].

The programme was designed to ensure that men with existing health conditions were not excluded. All men wishing to enrol in the pilot trial completed the Physical Activity Readiness Questionnaire (PAR-Q) [35]. Men answering ‘Yes’ to any question on the PAR-Q or whose measured blood pressure (BP) was at or over 140 mmHg (systolic) or 90 mmHg (diastolic) were advised to see their doctor before embarking on the programme.

### Ethics

The study was approved by the Ethics Committee of the School of Nursing, Midwifery and Health at the University of Stirling^a^. Participants gave written informed consent for participation in the pilot trial and randomization into either the intervention group (starting the programme within two weeks of the baseline measurements – the autumn 2010 delivery) or the waitlist comparison group (starting the programme four months later – the spring 2011 delivery). Men were offered travel expenses and a £20 football club shop voucher as a gesture of thanks for their participation in the follow up measurement sessions and focus group discussions.

### Intervention

The development, optimization and content of the FFIT programme is described in detail elsewhere [18]. In brief, the pilot programme (p-FFIT) was designed to be delivered by SPL club community coaching staff (mostly male sessional or full time coaches, with a broad range of qualifications and experience, who were employed by professional football clubs to deliver community activities) to groups of 15 men over twelve, 90 minute, weekly sessions at club home stadia. Each session comprised: a) classroom-based education focusing on topics related to successful weight management, such as healthy eating, reducing alcohol consumption and increasing daily physical activity; and b) coach-led physical activity sessions where men received training in aerobic, strength and flexibility exercises tailored to individual fitness levels, abilities and pre-existing health conditions (for more detail, see [18]). Men also undertook a daily incremental pedometer-based walking programme [36] to help them achieve 45 minutes of moderate physical activity on most days of the week, as recommended by national weight management guidance [37,38]. The dietary components were designed to deliver a 600 kcal/day deficit (from individual estimated daily energy requirements) [37,38].

p-FFIT provided instruction on the behaviour change techniques shown to be effective in physical activity and dietary interventions (e.g., self monitoring of weight and physical activity, specific goal setting, implementation intentions, feedback on behaviour) [39] and promoted peer and other forms of social support [39,40]. It also included components designed to appeal to male football supporters, including: club-based incentives (e.g., T-shirts in club colours); elements of competition (e.g., quizzes); an entire session focusing on alcohol consumption; and coach-led encouragement of the use of banter (e.g., football-related, often ironic or self-deprecatory jokes), thus actively facilitating the use of humour to help men address serious or sensitive topics (e.g., weight gain) that they may otherwise be reluctant to discuss with others [11,41-43].

### Comparison

All men (both intervention and comparison groups) received a standard information booklet containing weight loss advice [44] on enrolment in the pilot trial.

### Feasibility and acceptability

The primary outcomes for the pilot trial were feasibility and acceptability of the research procedures (including recruitment, randomization, data collection and retention). The feasibility and acceptability of the intervention, as assessed by participant feedback forms, participant focus group discussions and programme exit interviews, coach interviews, and direct observation of programme delivery sessions, is reported elsewhere [18].

Recruitment was assessed at baseline measurement sessions by asking men to report where they had heard about the programme. However, we were unable to report response rates, as the recruitment procedures used by the clubs did not permit estimation of the number of eligible men invited to take part. Participation in the follow-up measurements was used to assess retention through the trial. Attendance at the programme was obtained from coaches’ weekly attendance records. Acceptability of randomization was estimated from the percentage of men attending baseline measurements who gave informed consent to take part in the pilot trial.

A qualitative process evaluation was conducted, which included focus group discussions with intervention and comparison group participants following completion of the p-FFIT programme (four focus groups with a total of 26 men sampled purposively from a list of volunteers to include men of different ages and baseline BMIs). We used a semi-structured format to explore the feasibility and acceptability of the research procedures. Other issues addressed in the process evaluation (men’s views of the group-based delivery, programme components that men found useful/not useful for losing weight or becoming more active, suggestions for changes, coaches’ views of p-FFIT, and fidelity of delivery) are reported elsewhere [18].

The work described here was done to inform a fully-powered RCT (ISRCTN32677491), the primary objective of which is to determine whether FFIT (the optimized version of the pilot p-FFIT programme [18]) can help men achieve weight loss at least 5% greater than a waitlist comparison group 12 months after the start of their participation in the programme. Other outcomes include: weight loss at 12 weeks; changes in waist circumference, BP and percentage body fat at 12 weeks and 12 months; changes in self-reported physical activity, diet, alcohol consumption, self esteem, positive affect and health-related quality of life at 12 weeks and 12 months; short and long term cost-effectiveness; and process outcomes, including: fidelity of delivery; participant and coach experiences of involvement in p-FFIT; and participant experiences of maintaining weight loss and lifestyle changes over 12 months [30].

### Measurement

Outcome measurements for both the intervention and comparison groups were conducted at enrolment (baseline) and 12 weeks, and for the intervention group at 6 and 12 months. The comparison group did not take part in any follow up measurements beyond 12 weeks. Baseline, 12 week and 6 month assessments were undertaken at club stadia by members of the research team and fieldworkers fully trained by MRC/CSO Social and Public Health Sciences Unit Survey Office staff to standardised measurement and questionnaire administration protocols. Two in-stadia sessions were held at each time point in both clubs; questionnaires were sent out for self-completion to men who did not attend the stadia. In order to explore options for maximising retention, men who were unable to attend the stadia sessions at 12 months were given the opportunity to have a fieldworker visit them at home. If this was not practical or if they did not want a home visit, men were asked if they would be happy to have a questionnaire sent to them for self-completion. All men were contacted at each follow up time point, including those in the intervention group who did not complete the p-FFIT programme.

Weight (kg) was recorded using an electronic scale (Tanita HD 352), with participants wearing light clothing, no shoes and having emptied their pockets. In order to calculate BMI to assess eligibility to take part in the pilot trial, height (cm) was measured (without shoes) using a portable stadiometer (Seca Leicester). Waist circumference was obtained using a 200 cm tape measure to take at least two waist measurements (followed by a third if the first two differed by 5 mm or more). The mean of all recorded waist measurements was calculated for data analysis. Resting BP was measured using a digital BP monitor (Omron HEM-705CP), and body composition recorded (with participants lying down) using an electronic bioimpedance meter (BodyStat 1500 MDD). All equipment was calibrated prior to use.

Self-reported physical activity was assessed using the International Physical Activity Questionnaire (Short Form) (IPAQ) [45]. Self-reported diet was estimated using an adapted version of the Dietary Instrument for Nutrition Education (DINE) [46], which queried frequency of intake of 14 foods and drinks (cheese; beef burgers or sausages; beef, pork or lamb; fried food; chips; bacon or processed meat; pies, quiches or pastries; crisps; fast food; fruit and vegetables; chocolates or sweets; biscuits; sugary drinks; and milk) and frequency of breakfast consumption. DINE frequency categories were converted to scores as follows: no times/week = 0, 1–2 times/week = 1.5, 3–5 times/week = 4, 6 or more times/week = 6; less than once/day = 0.5, 1–2 times/day = 1.5, 3–5 times/day = 4, 6 or more times/day = 6; less than a quarter pint/day = 0, about a quarter pint/day = 0.25, about a half pint/day = 0.5, 1 pint or more/day = 1. Alcohol intake was estimated using a previous 7 days recall diary [47] and converted to units where a pint of beer or cider was scored as 2 units, a glass of wine as 1.5 units and a measure of spirits as 1 unit. Psychological outcomes were assessed using the Rosenberg Self Esteem Scale [48], the Positive and Negative Affect Scale (PANAS) [49] and the SF-12 [50]. Participant demographics (age, employment status, educational attainment, postcode, marital status, housing status and ethnicity) and how participants had heard about the programme were recorded at baseline only.

### Sample size

In order to test the feasibility of conducting a fully-powered RCT across 12 SPL clubs (where power calculations indicated an initial sample size of 360 men was required to detect a 5% difference in weight loss between the intervention and comparison groups at 12 months), a recruitment target of 60 was set for each club in the pilot trial. This target reflected the need to recruit 30 men to each arm of the trial (n=60) in every club to achieve the necessary numbers for the subsequent RCT.

### Randomization

Individual random assignment was determined using a computer-based random number sequence. The allocation ratio was 1:1, stratified by club.

### Quantitative and qualitative analyses

All statistical analyses were conducted using PASW Statistics 18 (SPSS Inc., Chicago, Illinois). Descriptive statistics (frequencies) were calculated for all baseline measures. Inferential statistics were used to test for differences in weight and other outcomes between baseline and 12 weeks for both intervention and comparison groups separately, and for between-group differences in change in weight and other outcomes from baseline to 12 weeks. Intention-to-treat analyses were used; specifically, all participants who provided data at each time point (including those who did not complete the p-FFIT programme) were analysed in the group they were allocated to. T-tests (paired or independent as appropriate) were conducted where data (or log transformed data) met assumptions of normality; otherwise non-parametric equivalents (Wilcoxon Signed Ranks or Mann Whitney tests) were carried out. Repeated-measures ANOVA or Friedman’s ANOVA were used to explore whether intervention group outcomes at 6 and 12 months were significantly different from baseline, with significant results followed up by post-hoc paired t-tests or Wilcoxon Signed Ranks tests. As this was a pilot trial and therefore hypothesis-generating, no corrections were made for multiple comparisons. Likewise, there was no generalised imputation for missing data. However, as the minimum change in weight over the course of the pilot trial and longer term follow up of the intervention group was of interest for the subsequent RCT (where weight loss at 12 months was the primary outcome), we used a highly conservative estimate for missing weight outcome data (baseline observation carried forward (BOCF)) to conduct sensitivity analyses. Data are presented as means ±SD or medians with IQ ranges: p values at/or below 0.05 were considered significant and are reported.

The focus group discussions were audio-recorded with participant consent and transcribed verbatim. Transcripts were analysed thematically using the Framework Approach [51], and NVivo9 software was used to assist data coding and organisation. The coding frame was based on our main research questions, but also allowed unanticipated themes to emerge and be systematically explored. Summary analyses of one theme, *Research procedures*, is relevant here. This theme included suggestions for alternative recruitment strategies and views of the randomization and measurement procedures. A subsample of transcripts (n=3) was cross-coded to verify high consistency of coding. Extracts from the focus group discussions are labeled to indicate group membership (“Int”= intervention group; “Comp”= comparison group) and participant identification number.

## Results

### Feasibility and acceptability – research procedures

Recruitment took place over a 3–4 week period in August and September 2010. Following consultation with club community coaching staff, different recruitment strategies were adopted in each setting. The city-based club used website advertising alone. The smaller club also posted leaflets to men who were on its season ticketholder database. Postcodes were examined to ensure that invitees lived locally to the club, but it was not possible to conduct any further screening for eligibility (i.e., age or BMI).

As Table 1 shows, the recruitment target was exceeded at the large, city-based club, but there was a slight shortfall at the smaller, town-based club. Club website advertising was the most effective recruitment strategy; sending leaflets to club season ticketholders was less productive. Despite this being the first time the programme had been delivered, news spread quickly and a number of men (particularly at the large club) reported hearing or receiving emails about the programme from third parties. Local and national media also picked up on the story, and some men reported reading about the programme in newspapers. A few men in each club had seen advertisements at their home ground or other local venues. Over a quarter of men reported hearing about the progamme from multiple (up to 5) sources.

**Table 1:** Summary of recruitment to the p-FFIT study

	**Large club**	**Smaller club**
Applied to join programme / Recruitment target	82 / 60	48 / 60
	**% (No)**	
Ineligible (age/BMI)	7.3 (6)	2.1 (1)
Withdrew for medical reasons	2.4 (2)	2.1 (1)
Changed mind	2.4 (2)	2.1 (1)
Not able to attend (programme or measurement sessions)	13.4 (11)	6.3 (3)
Randomized	30 Inter; 31 Comp^a^	21 Inter; 21 Comp
**Source** (where heard about programme)		
Club website	50.8 (31)	54.8 (23)
Leaflet mailings	n/a	19.0 (8)
Word of mouth (including emails)	44.3 (27)	28.6 (12)
Newspaper (local and national)	3.3 (2)	19.0 (8)
Other (e.g., adverts in local venues; match day advertising)	4.9 (3)	7.1 (3)
Men reporting more than one source	23.0 (14)	35.7 (15)

^a^Inter = intervention group; Comp = comparison group.

Focus group participants generally agreed that recruitment would have benefitted from the programme being advertised more widely:

Int 125: *A lot of folk were asking me how I actually got on the programme. I don’t think it was advertised enough, because a lot of my mates were like “Oh, how did you get on this?”*

At both clubs, men felt that more linking of publicity to home matches and other club activities would have increased interest in the programme:

Int 221: *To go wider, I think, people come to the game, obviously the* [match] *programme. I mean if you go to a lot of the local shops you see* [flyers advertising when] *the next game is*…

Int 213: *Put it on the flyer, on the flyer at the bottom.*

All men attending baseline enrolment sessions at their club stadia in September 2010 consented to being randomized to the intervention or comparison group. However, whilst men in the comparison group had generally found a four month delay in starting the programme acceptable, most raised concerns about the implications of having to wait 12 months before receiving the programme, as would be the case in the full RCT:

Comp 240: *As for waiting a year before you go on it – that’s too long, too long.*

Comp 234: *Some of the stuff circulated on the [online fans] forums and [wife] was involved in that. And some of the “you to you’s”* [messages] *she got back… it was almost heart breaking. Guys kind of saying, “This is my last chance”* […] A*nd I think them getting told, “Well, you need to wait a year”, I think that would be a bit of a blow.*

Comp 205: *I think it would put me off coming if I had to wait a year. I would just say “Och no, I’ll no’ bother then”, I wouldn’t be feeling up to it.*

Focus group participants were broadly comfortable with the measurement sessions, although specific concerns were raised about the time spent waiting between different measurement stations at baseline enrolment at one club, and difficulties with some of the wording in the questionnaires:

Int 208: *Probably the off-putting part was when you flipped to the back and seen how many questions you had to answer. And then some of them maybe you get stuck, and you’re thinking, “Am I reading this properly? Am I answering it the way they’re looking for it?”.*

**Figure 1:**
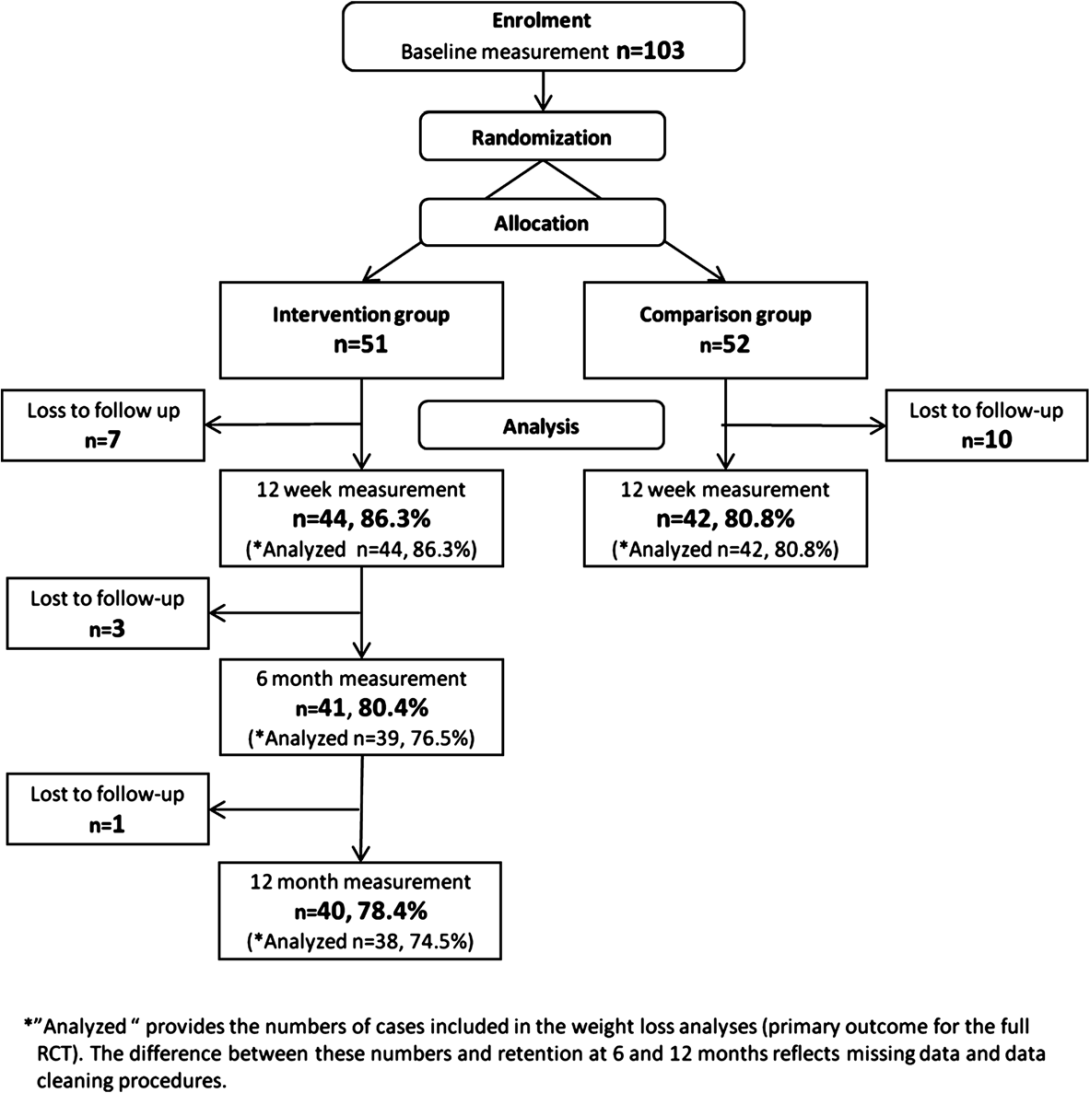
Participant flow through the p-FFIT study.

Retention through the study is shown in the CONSORT diagram (Figure 1). In-stadia 12 week measurements were conducted in December 2010; 6 month measurements in March 2011. At 12 months, 57% (29/51) of men in the intervention group attended the in-stadia measurements in September 2011, and a further 22% (11/51) were measured at home between October 2011 and January 2012. Three men withdrew from the study; one man from the intervention group died shortly after the 6 month measurements (of causes unrelated to involvement in the programme); fieldworkers were unable to establish contact with four men; and three men who did not want a home visit but agreed to having their questionnaire posted out for self-completion, did not return it.

In the intervention group, 76% (40/51) attended at least 80% of the available programme sessions (two sessions were cancelled in one club, and one session in the other club because of extreme winter weather conditions [52]). There were no marked differences in the age, baseline BMI, or baseline physical, lifestyle and psychological measures of men who stopped attending compared with those who kept coming.

### Baseline participant characteristics

A total of 103 men met the eligibility criteria for the pilot trial. As Table 2 shows, participants were recruited from across the socioeconomic spectrum, and all but one described their ethnic background as UK White. There were no differences between the groups according to baseline physical measures or demographic characteristics. The baseline values across a wide range of physical measures showed that the programme had succeeded in recruiting its target group of men who could benefit substantially from positive lifestyle changes. For example, mean BMI at baseline was 34.5 kg/m^2^: 45.6% had BMI>30 kg/m^2^, 30.1% had BMI>35 kg/m^2^ and 8.7% had BMI>40 kg/m^2^. Mean BP was 141.4 mmHg (systolic) and 90.9 mmHg (diastolic), and 68.0% had readings over the BP thresholds at which men were recommended to visit their GP (i.e., ≥140 mmHg systolic or ≥90 mmHg diastolic).

**Table 2:** Participant baseline characteristics: p-FFIT study

	**All**	**Intervention**	**Comparison**
**Physical measures**	**Mean±SD (No)**		
Age (years)	47.1±8.4 (103)	48.2±8.4 (51)	45.9±8.4 (52)
Weight (kg)	107.6±17.3 (103)	107.6±15.0 (51)	107.5±19.5 (52)
BMI (kg/m^2^)	34.5±5.0 (103)	34.5±3.9 (51)	34.5±6.0 (52)
Waist (cm)	116.9±10.9 (103)	117.2±9.6 (51)	116.5±12.1 (52)
Body Fat (%)	30.7±4.7 (90)	30.8±3.8 (42)	30.7±5.4 (48)
BP Systolic (mmHg)	141.4±15.6 (100)	142.7±17.8 (51)	140.1±13.1 (49)
BP Diastolic (mmHg)	90.5±10.3 (100)	89.8±8.9 (51)	91.3±11.7 (49)
**Employment status**	**% (No)**		
Full time work	76.7 (79)	76.5 (39)	76.9 (40)
Part time work	1.0 (1)	2.0 (1)	0.0 (0)
Unemployed	12.6 (13)	9.8 (5)	15.4 (8)
Student	1.9 (2)	3.9 (2)	0.0 (0)
Sick/disabled	2.9 (3)	3.9 (2)	1.9 (1)
Retired	4.9 (5)	3.9 (2)	5.8 (3)
**Educational attainment**	**% (No)**		
No qualifications	12.6 (13)	17.6 (9)	7.7 (4)
Standard grades or equivalent	19.4 (20)	17.6 (9)	21.2 (11)
Highers or equivalent	10.7 (11)	9.8 (5)	11.5 (6)
Vocational qualification	12.6 (13)	15.7 (8)	9.6 (5)
HNC/HND	16.5 (17)	7.8 (4)	25.0 (13)
First degree	16.5 (17)	23.5 (12)	9.6 (5)
Post-graduate qualification	8.7 (9)	7.8 (4)	9.6 (5)
Other	1.9 (2)	0.0 (0)	3.8 (2)
Missing	1.0 (1)	0.0 (0)	1.9 (1)
**Socioeconomic status**^**a**^	**% (No)**		
1 (most deprived)	16.5 (17)	15.7 (8)	17.3 (9)
2	20.4 (21)	19.6 (10)	21.2 (11)
3	20.4 (21)	21.6 (11)	19.2 (10)
4	18.4 (19)	17.6 (9)	19.2 (10)
5 (least deprived)	24.3 (25)	25.5 (13)	23.1 (12)
**Marital status**	**% (No)**		
Single	8.7 (9)	7.8 (4)	9.6 (5)
Married	71.8 (74)	76.5 (39)	67.3 (35)
Separated	4.9 (5)	5.9 (3)	3.8 (2)
Living with someone	10.7 (11)	5.9 (3)	15.4 (8)
Divorced	2.9 (3)	3.9 (2)	1.9 (1)
Widowed	1.0 (1)	0.0 (0)	1.9 (1)
**Housing status**	**% (No)**		
Own outright	20.4 (21)	25.5 (13)	15.4 (8)
Mortgage or loan	44.7 (46)	41.2 (21)	48.1 (25)
Part rent, part mortgage	1.0 (1)	0.0 (0)	1.9 (1)
Rent	30.1 (31)	27.5 (14)	32.7 (17)
Live rent free	2.9 (3)	3.9 (2)	1.9 (1)
Other	1.0 (1)	2 (1)	0.0 (0)
**Ethnicity**	**% (No)**		
White UK	99.0 (102)	100.0 (51)	98.1 (51)
Mixed Race	1.0 (1)	0.0 (0)	1.9 (1)

^a^Estimated using the Scottish Index of Multiple Deprivation based on home postcode (http://www.scotland.gov.uk/Topics/Statistics/SIMD).

### Changes in outcomes from baseline to 12 weeks

#### Physical measures

As Figure 1 shows, 86% (44/51) men in the intervention group and 81% (42/52) men in the comparison group took part in the 12 week measurements. These figures were achieved despite extreme winter weather conditions in December 2010 [52], which severely restricted fieldworker and participant travel to the in-stadia measurement sessions. This meant that in the larger club we had to prioritise collecting weight and waist circumference measures above BP and body composition measures at the main in-stadia session, and subsequently post questionnaires to men for self-completion. All data were collected as per protocol in the other club.

Table 3 provides the estimated impact of the intervention on weight and other physical measures (waist circumference, BP, body composition (percentage body fat)). The intervention group lost 4.6 ±2.8% (SD) of their baseline weight during the 12 week programme (p<.001), whilst the comparison group gained 0.6 ±0.2% (n.s) (between-group difference p<.001). Sensitivity analyses using BOCF to provide a conservative estimate of 12 week weight loss for all participants were also highly significant (baseline-12 week weight change: intervention group p<.001, comparison group n.s.; between-group difference p<.001). As Table 4 shows, almost half (45.5%) of intervention group participants achieved a clinically-significant weight loss of at least 5% at 12 weeks compared to none of the comparison group. Participants in the intervention group also showed significant reductions in waist circumference (p<.001) and systolic BP (p=.013) compared to the comparison group (see Table 3).

**Table 3:** Physical measures at baseline, 12 weeks, 6 months and 12 months: p-FFIT study

	**Group**^**a**^	**Baseline**	**12 weeks**	**Between-group difference in change between baseline and 12 weeks**	**6 months**	**12 months**
		**Mean**^**(sig p-value)**^ **± SD (No)**			**Mean**^**(sig p-value)**^ **± SD (No)**	
Weight (kg)	Inter	**107.6** ± 15.0 (51)	**101.6**^**(<.001)**^ ± 14.1 (44)	**<.001**	**101.7**^**(<.001)**^ ± 13.2 (39)	**102.0**^**(<.001)**^ ± 13.2 (38)
	Comp	**107.5** ± 19.5 (52)	**106.2** ± 18.5 (42)			
Weight (kg) (BOCF)	Inter		**103.3**^**(<.001)**^ ± 14.3 (51)	**<.001**	**103.1**^**(<.001)**^ ± 13.2 (51)	**104.8**^**(<.001)**^ ± 14.8 (51)
	Comp		**108.0** ± 19.0 (52)			
Weight loss from baseline (%)	Inter	n/a	**4.6** ± 2.8 (44)	**<.001**	**5.2** ± 4.2 (39)	**3.5** ± 4.8 (38)
	Comp	n/a	**−0.6** ± 2.0 (42)			
Weight loss from baseline (%) (BOCF)	Inter		**3.9** ± 3.0 (51)	**<.001**	**4.0** ± 4.3 (51)	**2.6** ± 4.4 (51)
	Comp		**−0.5** ± 1.8 (52)			
Waist circumference (cm)	Inter	**117.2** ± 9.6 (51)	**113.5**^**(<.001)**^ ± 9.9 (44)	**<.001**	**110.5**^**(<.001)**^ ± 10.1 (34)	**112.0**^**(<.001)**^ ± 9.0 (39)
	Comp	**116.5** ± 12.1 (52)	**116.7**^**(.014)**^ ± 12.9 (41)			
Systolic BP (mmHg)	Inter	**142.7** ± 17.8 (51)	**131.8**^**(.003)**^ ± 17.5 (24)	**.013**	**134.2** ± 16.4 (30)	**139.0** ± 19.3 (38)
	Comp	**140.1** ± 13.1 (49)	**138.2** ± 19.1 (26)			
Diastolic BP (mmHg)	Inter	**89.8** ± 8.9 (51)	**81.9**^**(<.001)**^ ± 8.4 (24)		**86.8** ± 11.1 (30)	**85.2**^**(.003)**^ ± 9.6 (38)
	Comp	**91.3** ± 11.7 (49)	**86.9** ± 12.7 (26)			
Body fat (%)	Inter	**30.8** ± 3.8 (42)	**29.7** ± 3.7 (25)	**<.001**	**29.7**^**(.019)**^ ± 4.2 (31)	**29.4**^**(.003)**^ ± 4.0 (35)
	Comp	**30.7** ± 5.4 (48)	**32.8**^**(<.001)**^ ± 5.6 (26)			

Note. Intervention group measured at baseline, 12 weeks, 6 months and 12 months; comparison group measured at baseline and 12 weeks only. Significant p-values (sig p-value) are reported for before-and-after within-group differences at 12 weeks, 6 months and 12 months, and between-group differences at 12 weeks. Sample sizes vary due to missing values. Sensitivity analyses conducted on weight outcomes using baseline observation carried forward (BOCF).

^a^Inter = intervention group; Comp = comparison group.

**Table 4:** Percentage weight loss at 12 weeks, 6 months and 12 months: p-FFIT study

**Group**^**a**^	**Outcome**	**12 weeks**	**6 months**	**12 months**
		**% (No)**		
		n=44	n=39	n=38
Inter	Gained weight	2.3 (1)	12.8 (5)	21.1 (8)
	Stable (±0.5 kg)	2.3 (1)	2.6 (1)	5.3 (2)
	Lost up to 5%	50.0 (22)	28.2 (11)	34.2 (13)
	Lost 5-10%	43.2 (19)	43.6 (17)	31.6 (12)
	Lost more than 10%	2.3 (1)	12.8 (5)	7.9 (3)
	**Men losing at least 5%**	**45.5 (20)**	**56.4 (22)**	**39.5 (15)**
		n=42		
Comp	Gained weight	52.4 (22)		
	Stable (±0.5 kg)	19.0 (8)		
	Lost up to 5%	28.6 (12)		
	Lost 5-10%	0.0 (0)		
	Lost more than 10%	0.0 (0)		
	**Men losing at least 5%**	**0.0 (0)**		

^a^Inter = intervention group; Comp = comparison group.

#### Lifestyle measures

Table 5 shows men’s self-reported physical activity. Over the course of the 12 week programme, the intervention group reported marked increases in total, vigorous and moderate activity, whilst the comparison group did not (between-group differences: total activity p=.001; vigorous activity p=.014; moderate activity p<.001).

**Table 5:** Physical activity outcomes at baseline, 12 weeks, 6 months and 12 months: p-FFIT study

	**Group**^**a**^	**Baseline**	**12 weeks**	**Between-group difference in change between baseline and 12 weeks**	**6 months**	**12 months**
**Self-reported physical activity: median**^**(sig p-value)**^**IQ range (No)**						
Total activity (MET min/week)	Inter	**1188** 475-1971 (42)	**2840**^**(.001)**^ 1873-5532 (33)	**.001**	**3434** 1579-5220 (26)	**1866** 968-5946 (11)
	Comp	**1307** 396-2937 (39)	**1055** 330-2346 (27)			
Vigorous activity (MET min/week)	Inter	**0** 0-360 (49)	**960**^**(.001)**^ 160-2880 (37)	**.014**	**1200**^**(.012)**^ 0-1980 (34)	**960**^**(.005)**^ 0-2520 (34)
	Comp	**0** 0-960 (47)	**0** 0-960 (33)			
Moderate activity (MET min/week)	Inter	**0** 0-80 (47)	**360**^**(<.001)**^ 0-1860 (37)	**<.001**	**480**^**(.001)**^ 0-1360 (33)	**240**^**(.002)**^ 0-1440 (35)
	Comp	**0** 0-480 (46)	**0** 0-180 (32)			
Walking (MET min/week)	Inter	**693** 259-1337 (46)	**990** 495-1832 (38)		**1040** 396-2079 (36)	**924** 495-1782 (33)
	Comp	**462** 264-1386 (43)	**495** 272-1386 (34)			
**Self-reported sedentary behaviour: median**^**(sig p-value)**^**IQ range (No)**						
Sitting time (hours)	Inter	**7.0** 4.0-10.0 (42)	**6.0**^**(.026)**^ 4.0-8.0 (31)		**6.0**^**(.003)**^ 4.1-7.4 (36)	**5.0**^**(.014)**^ 4.0-7.4 (32)
	Comp	**8.0** 5.0-11.0 (44)	**8.0** 5.3-11.5 (32)			

Note. Intervention group measured at baseline, 12 weeks, 6 months and 12 months; comparison group measured at baseline and 12 weeks only. Significant p-values (sig p-value) are reported for before-and-after within-group differences at 12 weeks, 6 months and 12 months, and between-group differences at 12 weeks. Sample sizes vary due to missing values.

^a^Inter = intervention group; Comp = comparison group.

Table 6 shows self-reported dietary habits. Compared to the comparison group, the intervention group reported significant improvements in diet including: increased frequency of eating breakfast (p=.004) and fruit and vegetables (p=.01); and decreased frequency of eating bacon or processed meats (p=.01), crisps (p=.05), chocolates or sweets (p=.037) and biscuits (p=.008). Self-reported alcohol consumption at 12 weeks remained similar in both groups (Table 7).

**Table 6:** Dietary outcomes at baseline, 12 weeks, 6 months and 12 months: p-FFIT study

	**Group**^**a**^	**Baseline**	**12 weeks**	**Between group difference in change between baseline and 12 weeks**	**6 months**	**12 months**
		**Mean**^**(sig p-value)**^ **± SD (No)**			**Mean**^**(sig p-value)**^ **± SD (No)**	
Breakfast (times/week)	Inter	**4.6** ± 1.9 (51)	**5.6**^**(.002)**^ ± 0.8 (39)	**.004**	**5.5**^**(.005)**^ ± 0.9 (40)	**5.4** ± 1.2 (39)
	Comp	**4.2** ± 2.2 (52)	**4.4** ± 2.2 (38)			
Cheese (times/week)	Inter	**2.3** ± 1.7 (49)	**1.7**^**(.044)**^ ± 1.4 (39)		**1.7** ± 1.2 (40)	**2.3** ± 1.7 (40)
	Comp	**2.5** ± 1.6 (51)	**2.6** ± 1.7 (38)			
Beef burgers or sausages(times/week)	Inter	**1.4** ± 1.0 (51)	**0.8**^**(.002)**^ ± 0.8 (39)		**1.1** ± 0.8 (39)	**1.0** ± 0.9 (39)
	Comp	**1.4** ± 1.2 (52)	**1.2** ± 0.8 (37)			
Beef, pork or lamb (times/week)	Inter	**2.4** ± 1.5 (51)	**2.1** ± 1.4 (39)		**2.3** ± 1.2 (40)	**2.0** ± 1.4 (40)
	Comp	**2.1** ± 1.3 (52)	**2.0** ± 1.4 (38)			
Fried food (times/week)	Inter	**1.3** ± 1.1 (50)	**1.0** ± 1.2 (37)		**0.9** ± 0.9 (40)	**0.9** ± 0.7 (40)
	Comp	**1.8** ± 1.7 (52)	**1.5** ± 1.5 (37)			
Chips (times/week)	Inter	**1.9** ± 1.4 (50)	**1.3**^**(.038)**^ ± 1.4 (39)		**1.1**^**(.005)**^ ± 1.0 (40)	**1.1**^**(.001)**^ ± 0.8 (39)
	Comp	**1.8** ± 1.6 (52)	**1.7** ± 1.4 (38)			
Bacon or processed meats (times/week)	Inter	**1.8** ± 1.4 (51)	**1.1** ± 1.1 (38)	**.010**	**1.5** ± 1.3 (40)	**1.5** ± 1.5 (39)
	Comp	**1.7** ± 1.4 (52)	**1.9** ± 1.3 (38)			
Pies, quiches or pastries (times/week)	Inter	**1.3** ± 1.1 (50)	**0.9**^**(.040)**^ ± 0.7 (38)		**1.0** ± 1.2 (40)	**0.8** ± 0.8 (39)
	Comp	**1.2** ± 1.0 (52)	**1.5** ± 1.1 (38)			
Crisps (times/week)	Inter	**2.3** ± 2.0 (50)	**1.1**^**(.003)**^ ± 1.3 (38)	**.050**	**2.0** ± 1.8 (40)	**1.8** ± 1.5 (40)
	Comp	**2.4** ± 2.0 (52)	**2.0** ± 2.0 (38)			
Fast foods (times/week)	Inter	**1.1** ± 1.3 (51)	**0.8** ± 0.8 (39)		**0.9** ± 0.9 (40)	**0.8** ± 0.8 (40)
	Comp	**1.5** ± 1.4 (51)	**1.0** ± 1.0 (38)			
Fruit and vegetables (times/day)	Inter	**2.1** ± 1.7 (51)	**3.9**^**(<.001)**^ ± 1.8 (39)	**.010**	**3.5**^**(<.001)**^ ± 1.7 (40)	**3.5**^**(<.001)**^ ± 1.6 (40)
	Comp	**1.7** ± 1.4 (52)	**2.4**^**(.023)**^ ± 1.7 (38)			
Chocolates or sweets (times/day)	Inter	**1.6** ± 1.3 (51)	**0.8**^**(<.001)**^ ± 0.4 (39)	**.037**	**0.8**^**(.001)**^ ± 0.7 (40)	**0.9**^**(.008)**^ ± 0.5 (40)
	Comp	**1.7** ± 1.6 (52)	**1.5** ± 1.4 (38)			
Biscuits (times/day)	Inter	**1.7** ± 1.3 (50)	**0.9**^**(.001)**^ ± 0.8 (39)	**.008**	**1.2** ± 1.0 (40)	**1.1**^**(.003)**^ ± 0.8 (40)
	Comp	**1.6** ± 1.4 (52)	**1.7** ± 1.4 (38)			
Sugary drinks (times/day)	Inter	**1.7** ± 1.7 (51)	**1.3** ± 1.7 (39)	**.001**	**1.2** ± 1.5 (40)	**1.5** ± 1.5 (40)
	Comp	**1.7** ± 1.8 (52)	**2.3**^**(.006)**^ ± 2.1 (38)			
Milk (pints/day)	Inter	**0.4** ± 0.3 (51)	**0.4** ± 0.3 (39)		**0.3** ± 0.3 (40)	**0.4** ± 0.4 (40)
	Comp	**0.5** ± 0.4 (52)	**0.4** ± 0.3 (38)			

Note. Intervention group measured at baseline, 12 weeks, 6 months and 12 months; comparison group measured at baseline and 12 weeks only. Significant p-values (sig p-value) are reported for before-and-after within-group differences at 12 weeks, 6 months and 12 months, and between-group differences at 12 weeks. Sample sizes vary due to missing values.

^a^Inter = intervention group; Comp = comparison group.

**Table 7:** Alcohol consumption (units per week) at baseline, 12 weeks, 6 months and 12 months: p-FFIT study

	**Group**^**a**^	**Baseline**	**12 weeks**	**Between group difference in change between baseline and 12 weeks**	**6 months**	**12 months**
		**Mean**^**(sig p-value)**^** ± SD (No)**			**Mean**^**(sig p-value)**^** ± SD (No)**	
Beer and cider	Inter	**11.6** ± 12.0 (50)	**8.3** ± 10.6 (39)		**7.9**^**(.021)**^ ± 12.5 (41)	**7.2**^**(.007)**^ ± 9.8 (40)
	Comp	**10.3** ± 14.3 (51)	**6.6** ± 7.9 (38)			
Wine	Inter	**4.6** ± 9.2 (50)	**3.9** ± 6.5 (39)		**3.2** ± 5.2 (41)	**3.4** ± 7.1 (40)
	Comp	**4.5** ± 9.7 (51)	**4.8** ± 9.1 (38)			
Spirits	Inter	**1.2** ± 3.7 (50)	**1.9** ± 3.7 (39)		**1.6** ± 3.3 (41)	**1.3** ± 3.5 (40)
	Comp	**2.2** ± 6.0 (51)	**2.3** ± 4.0 (38)			
Total alcohol	Inter	**17.4** ± 16.5 (50)	**14.1** ± 13.6 (39)		**12.6**^**(.016)**^ ± 13.8 (41)	**11.9**^**(.041)**^ ± 12.3 (40)
	Comp	**16.9** ± 16.3 (51)	**13.6** ± 12.5 (38)			

Note. Intervention group measured at baseline, 12 weeks, 6 months and 12 months; comparison group measured at baseline and 12 weeks only. Significant p-values (sig p-value) are reported for before-and-after within-group differences at 12 weeks, 6 months and 12 months, and between-group differences at 12 weeks. Sample sizes vary due to missing values.

^a^Inter = intervention group; Comp = comparison group.

#### Psychological measures

Table 8 shows self-reported psychological measures. The intervention group recorded a significant improvement in self esteem at 12 weeks compared to the comparison group (p=.002).

**Table 8:** Psychological measures at baseline, 12 weeks, 6 months and 12 months: p-FFIT study

	**Group**^**a**^	**Baseline**	**12 weeks**	**Between group difference in change between baseline and 12 weeks**	**6 months**	**12 months**
		**Mean**^**(sig p-value)**^** ± SD (No)**			**Mean**^**(sig p-value)**^** ± SD (No)**	
Self esteem	Inter	**19.2** ± 4.3 (50)	**22.5**^**(<.001)**^ ± 3.5 (39)	**.002**	**22.5**^**(<.001)**^ ± 4.2 (40)	**22.6**^**(<.001)**^ ± 3.7 (40)
	Comp	**18.7** ± 4.1 (52)	**19.6** ± 4.4 (37)			
Positive affect	Inter	**16.8** ± 3.1 (50)	**19.0**^**(.001)**^ ± 2.2 (39)		**18.4**^**(<.001)**^ ± 2.7 (41)	**17.8**^**(.032)**^ ± 3.6 (40)
	Comp	**16.0** ± 2.9 (49)	**16.7** ± 3.3 (38)			
Negative affect	Inter	**9.3** ± 3.0 (50)	**8.7** ± 3.3 (39)		**8.6** ± 3.0 (41)	**8.9** ± 3.3 (40)
	Comp	**9.3** ± 3.1 (49)	**9.4** ± 3.1 (38)			
SF-12 Physical	Inter	**49.0** ± 6.8 (51)	**49.5** ± 8.6 (39)		**51.3** ± 7.3 (41)	**52.0** ± 5.7 (40)
	Comp	**48.1** ± 7.6 (51)	**49.2** ± 6.9 (38)			
SF-12 Mental	Inter	**49.2** ± 10.3 (51)	**54.4**^**(.003)**^ ± 8.0 (39)		**52.7** ± 7.2 (41)	**52.9**^**(.022)**^ ± 5.7 (40)
	Comp	**47.1** ± 9.5 (51)	**48.8** ± 9.0 (38)			

Note. Intervention group measured at baseline, 12 weeks, 6 months and 12 months; comparison group measured at baseline and 12 weeks only. Significant p-values (sig p-value) are reported for before-and-after within-group differences at 12 weeks, 6 months and 12 months, and between-group differences at 12 weeks. Sample sizes vary due to missing values.

^a^Inter = intervention group; Comp = comparison group.

### Changes in intervention group outcomes from baseline to 6 and 12 months

#### Physical measures

As Figure 1 shows, over 80% (41/51) of men in the intervention group took part in the measurements at 6 months, and over 78% (40/51) at 12 months. Table 3 shows that reductions in weight remained significant at 6 months and 12 months (both p<.001). Sensitivity analyses using BOCF estimates of missing weight data were also highly significant at both time points (both p<.001). These figures equate to over half (56.4%) of participants achieving a clinically-significant (at least 5%) weight loss at 6 months, and almost 40% at 12 months (shown in Table 4). Reductions in waist circumference also remained significant at 6 months and 12 months (both p<.001) (Table 3).

#### Lifestyle measures

As Table 5 shows, the intervention group’s self-reported physical activity remained significantly higher than baseline at 6 months (vigorous activity p=.012; moderate activity p=.001) and 12 months (vigorous activity p=.005; moderate activity p=.002). Men also reported less time spent sitting at 6 months (p=.003) and 12 months (p=.014) than at baseline. Significant improvements in self-reported diet (shown in Table 6) were sustained to 6 months (increased frequency of eating breakfast (p=.005) and fruit and vegetables (p<.001); decreased frequency of eating chocolates or sweets (p=.001)) and 12 months (increased frequency of eating fruit and vegetables (p<.001); decreased frequency of eating chocolates or sweets (p=.008) and biscuits (p=.003)). Men also reported eating fewer chips at 6 months (p=.005) and 12 months (p=.001). Finally, Table 7 demonstrates that there were significant reductions from baseline in self-reported beer and cider consumption over the longer term (6 months p=.021; 12 months p=.007).

#### Psychological measures

Men in the intervention group continued to report highly significant improvements in self-esteem 6 and 12 months after starting the programme (both p<.001) (Table 8). Positive affect was also increased at 6 months (p<.001) and 12 months (p=.032).

## Discussion

This pilot randomized trial was undertaken to assess the feasibility of conducting a fully-powered randomized controlled trial (RCT) of 12 month weight loss in the professional football club setting, and to inform the final design and research procedures for the full RCT. Specifically, it aimed to examine the feasibility and acceptability of the recruitment, randomization and measurement procedures, and to provide estimates of: retention to 12 months (in the intervention group only); weight loss at 12 weeks retained to 12 months; and changes in other physical biomarkers of health risk (e.g., waist circumference and BP) and self-reported lifestyle and psychological measures at 12 weeks and 12 months (secondary outcomes in the RCT). The results confirmed that recruitment and retention were adequate to proceed to the full RCT (with some modifications to planned recruitment strategies and research procedures) and that the intervention showed potential to support men in losing weight and making positive changes to other physical biomarkers of health risk, lifestyle behaviours and psychological outcomes.

### Feasibility and acceptability

The challenges of conducting robust evaluations of interventions delivered through professional sports club settings have been recognized [23,24]. Previous studies have either been small scale [20,22] or have had to compromise a rigorous systematic approach to the constraints of working with community-based partners [21,26-28]. In a study assessing the impact and uptake of a range of health-related activities delivered through English Premier League clubs, Pringle and colleagues [21] noted that the use of self-reported rather than objective measures and non-independent methods of data collection are “common [issues] in evaluating community lifestyle interventions, and highlight an important distinction between research reflecting clinical standards and evaluation concerned with yielding practice-based evidence” (p415). Like others, we had good reason to wonder, prior to conducting this pilot trial, whether the methods and measures required for a randomized controlled trial in this type of community setting would be acceptable to individual men and to the football clubs. The lessons learned from the current study will be therefore be extremely valuable for informing future research conducted in professional football clubs and other similar settings.

Despite the short (3–4 week) recruitment period, high levels of interest led to word of the programme spreading rapidly, particularly in the large club. The high proportion of overweight/obese men with elevated BP readings at baseline demonstrated that the recruitment strategies and football club setting were successful in targeting at-risk men. Slower recruitment in the smaller club suggested the need for more intensive recruitment strategies to be implemented in some clubs during the RCT; multiple prompts may also be required. Although season ticketholder databases have previously been used successfully to recruit participants to studies in large football clubs [22], the pilot trial demonstrated that this strategy may be less effective in smaller clubs. More productive strategies might include linking advertising to home matches and other club activities, and promoting word of mouth (particularly using former participants to act as credible role models who the target population can identify with [11,18,53]). In order to maximize recruitment to the full RCT, if participant numbers remain below target in clubs with smaller fanbases, the recruitment figures for the large club suggest that it may also be possible to ask clubs with larger fanbases to deliver additional programmes.

The recruitment of men from across the socioeconomic spectrum, without any specific targeting of those from areas of higher deprivation, provides further support for the view that professional football clubs can help to address health inequalities by encouraging population groups at increased risk of ill health to engage in organized health promotion activities [21]. However, p-FFIT failed to address the under-representation of men from ethnic minorities in weight management programmes [9]. Whilst it is important to remember that many SPL football clubs are based in areas which are much more ethnically uniform than other parts of the UK, only 1 out of 103 participants described himself as being non-White. Additional work is therefore required to understand why p-FFIT did not attract men from minority ethnic groups and what changes/adaptations might be required to increase engagement (e.g., building links with local religious communities [54]).

Although randomization to start p-FFIT immediately or after a four month delay was shown to be feasible and broadly acceptable to participants (including those in the comparison group), there was some concern about the prospect of having to wait 12 months before starting the programme. As a 12 month delay for the comparison group is unavoidable in the full RCT, it will be essential to ensure that participants feel their contribution to the research is valued by offering vouchers and travel expenses at follow up measurements, and by taking a personalized approach (e.g., individualized letters and telephone calls) at all research contacts in order to maximize retention.

Issues with extended waiting times at some (but not all) measurement sessions and with comprehension of some parts of the questionnaire underline the importance of providing adequate staffing at in-stadia measurements. Sufficient staff should be rostered for each session to ensure smooth progression of men through the measurement stations, and fieldworkers given full training in assisting men with questionnaire completion if required (e.g., if participants have literacy problems). Despite these problems, retention through the pilot trial remained high. Follow up rates met criteria (less than 20% attrition at 12 weeks and 6 months, and less than 30% attrition at 12 months) that have been cited as acceptable for weight loss and lifestyle change interventions [25,55]. The strategy of offering follow up home visits at 12 months was extremely effective: it reduced attrition by almost half (from 43.1% to 21.6%), thus minimising bias in the study outcomes [56]. This finding supports investment in home visits at all follow up measurement time points during the full RCT.

### Health and behavioural outcomes

Although the pilot trial was not powered to detect between-group differences in health, lifestyle and psychological outcomes, a number of significant results were observed at 12 weeks, many of which were maintained in the intervention group to 6 and 12 months. These give an indication of the intervention’s potential effectiveness. The difference in percentage weight loss between the intervention and comparison groups (5.2%) was comparable with the results of a meta-analysis of weight loss outcomes from previous RCTs of men-only weight loss interventions, which reported a 5.7% between-group difference in weight loss at the last reported assessment [25]. Individually these RCTs reported 1.0-6.3 kg between-group weight loss differences at 3 months [15,54,57,58], 5.3-7.6 kg before-and-after weight loss at 6 months [54,57], and 2.6-6.7 kg before-and-after weight loss at 12 months [59-61].

### Limitations

Although the pilot trial demonstrated significant differences in weight and other outcomes between the intervention and comparison groups at 12 weeks, these are clearly indicative rather than definitive. The sample was intentionally small, as appropriate for a pilot study, and drawn from two out of the twelve clubs in the Scottish Premier League, meaning that no conclusions can be drawn about the generalisablity of the findings. Lack of power means it is also important not to over-interpret a lack of statistical significance in between-group differences.

The comparison group received the programme just four months after the intervention group. We were therefore unable to compare intervention group 12 month outcomes with a group who had not taken part in the programme. This makes it difficult to draw firm conclusions about the programme’s likely longer term impact and about comparison group retention to 12 months. However, over 80% of comparison group men took part in the 12 week measures (including five men who agreed to participate in the pilot trial despite no longer being able to take part in the programme), suggesting that longer term comparison group retention is likely to be adequate.

The fact that website advertising was the main source of recruitment to the pilot trial, meant that we were unable to calculate intervention reach [62]. It had been hoped that season ticketholder databases could be used to provide an estimation of response rate, however absence of information on age and BMI in these databases made it impossible to get an accurate figure for the total number of potential participants (i.e., men who were aged between 35–65 years with BMI≥27 kg/m^2^, and thus eligible to take part in the pilot trial).

Physical activity, diet and alcohol consumption were assessed through self-report. Although more objective measurement (e.g., accelerometry, interviewer-administered recall) might be considered desirable, this would be logistically extremely difficult and prohibitively expensive to collect in the fully-powered RCT. As these are secondary outcomes in the RCT, a pragmatic decision was taken that self-report would be adequate to provide an estimate of change over time, recognizing the potential for response bias (e.g., inaccurate recall, social desirability) [63]. Future work should assess the acceptability of including these more time-consuming and potentially intrusive measures in the evaluation of interventions delivered in this setting.

Participants were individually randomised within club, raising the possibility of contamination between the intervention and comparison groups at each club and the issue of whether cluster randomization would be more appropriate. However, for contamination to be a real problem, it would have to be assumed that discussion of the intervention between peers (i.e., men in the comparison group talking to their counterparts in the intervention group) is as effective as receiving the full intervention from professional community coaches trained in the programme delivery protocol. This is unlikely to be the case, and therefore cluster randomization is unwarranted [64].

Finally, as men in the comparison group enrolled in the study in the hope that they would be able to access the p-FFIT intervention immediately, the fact that they had to wait 4 months before receiving it will have likely led to feelings of disappointment. Some may have decided to seek an alternative intervention or to try to lose weight independently (compensatory rivalry). The fact that some comparison group participants did lose a minimal amount of weight between baseline and 12 weeks suggests that some compensatory rivalry may have taken place. However, as none achieved 5% weight loss, any impact of compensatory rivalry appears to have been minimal and furthermore would mean that the reported between-group differences are slightly conservative estimates of intervention effectiveness.

## Conclusions

The findings of this pilot randomized trial support the conduct of a fully-powered randomized controlled trial (RCT) across all Scottish Premier League clubs to evaluate the effectiveness of the Football Fans in Training (FFIT) intervention in helping men achieve a clinically-significant weight loss that is maintained to 12 months [30]. Recruitment and retention rates were adequate. The randomization and measurement procedures and the intervention itself [18] were broadly acceptable to participants, but some minor modifications to protocol were identified as necessary to ensure the successful conduct of the RCT. The study also suggested that FFIT has potential to support men in losing weight and making positive lifestyle changes, some of which are maintained in the longer term.

## Endnote

^a^CG and SW moved to the University of Glasgow from the University of Stirling in 2011.

## Competing interests

The authors declare that they have no competing interests.

## Authors’ contributions

CMG, KH, NM, ASA, ST and SW contributed to the research design. CMG, KH and SW assisted with data collection at the club stadia. KH, NM, ASA, ST and SW provided support and guidance during data analysis. CMG performed the statistical analyses, conducted the focus group discussions, supported the research assistant performing the qualitative analyses, and drafted the manuscript. All authors commented on drafts, and read and approved the final manuscript.

## References

[B1] Finucane MM Stevens GA Cowan MJ Danaei G Lin JK Paciorek CJ National, regional, and global trends in body-mass index since 1980: systematic analysis of health examination surveys and epidemiological studies with 960 country-years and 9.1 million participants Lancet 2012 10 557 567 10.1016/S0140-6736(10)62037-5 PMC4472365 21295846

[B2] NHS Information Centre Health survey for England 2011: health, social care and lifestyles 2012 Leeds: The Health and Social Care Information Centre

[B3] Scottish Government Scottish health survey 2011 2012 Edinburgh: Scottish Government

[B4] Government Office for Science Foresight tackling obesities: future choices - project report 2nd edition 2007 London: Department of Innovation Universities and Skills

[B5] Bye C Avery A Lavin J Tackling obesity in men: preliminary evaluation of men-only groups within a commercial slimming organization J Hum Nutr Diet 2005 10 391 394 10.1111/j.1365-277X.2005.00642.x 16150135

[B6] Counterweight Project Team Evaluation of the Counterweight programme for obesity management in primary care: a starting point for continuous improvement Br J Gen Pract 2008 10 548 554 10.3399/bjgp08X319710 18682018 PMC2486382

[B7] Jolly K Lewis A Beach J Denley J Adab P Deeks JJ Comparison of range of commercial or primary care led weight reduction programmes with minimal intervention control for weight loss in obesity: Lighten Up randomised controlled trial Br Med J 2011 10 doi:10.1136/bmj.d6500 10.1136/bmj.d6500 PMC3208022 22053315

[B8] Jebb SA Ahern AL Olson AD Aston LM Holzapfel C Stoll J Primary care referral to a commercial provider for weight loss treatment versus standard care: a randomised controlled trial Lancet 2011 10 1485 1492 10.1016/S0140-6736(11)61344-5 21906798 PMC3207352

[B9] Pagoto SL Schneider KL Oleski JL Luciani JM Bodenlos JS Whited MC Male inclusion in randomized controlled trials of lifestyle weight loss interventions Obesity 2012 10 1234 1239 10.1038/oby.2011.140 21633403

[B10] Wardle J Johnson F Weight and dieting: examining levels of weight concern in British adults Int J Obesity 2002 10 1144 1149 10.1038/sj.ijo.0802046 12119582

[B11] Sabinsky MS Toft U Raben A Holm L Overweight men's motivations and perceived barriers towards weight loss Eur J Clin Nutr 2007 10 526 531 16988645 10.1038/sj.ejcn.1602537

[B12] Stibbe A Health and the social construction of masculinity in Men's Health magazine Men and Masculinities 2004 10 31 51 10.1177/1097184X03257441

[B13] Cafri G Thompson JK Measuring male body image: a review of the current methodology Psychol Men Masculinity 2004 10 18 29

[B14] Gough B Conner MT Barriers to healthy eating amongst men: a qualitative analysis Soc Sci Med 2006 10 387 395 10.1016/j.socscimed.2005.05.032 16011867

[B15] Morgan PJ Collins CE Plotnikoff RC Cook AT Berthon B Mitchell S Efficacy of a workplace-based weight loss program for overweight male shift workers: the workplace POWER (Preventing Obesity Without Eating like a Rabbit) randomized controlled trial Prevent Med 2011 10 317 325 10.1016/j.ypmed.2011.01.031 21300083

[B16] Gough B 'Real men don't diet': an analysis of contemporary newspaper representations of men, food and health Soc Sci Med 2007 10 326 337 10.1016/j.socscimed.2006.09.011 17070972

[B17] Kiefer I Rathmanner T Kunze M Eating and dieting differences in men and women J Men's Health Gender 2005 10 194 201

[B18] Gray CM Hunt K Mutrie N Anderson AS Leishman J Dalgarno L Football Fans in Training: the development and optimization of an intervention delivered through professional sports clubs to help men lose weight, become more active and adopt healthier eating habits BMC Public Health 2013 10 doi:10.1186/1471-2458-13-232 10.1186/1471-2458-13-232 PMC3621078 23496915

[B19] Hirt ER Clarkson JJ Kahle LR, Close AG The psychology of fandom: understanding the etiology, motives, and implications of fanship Consumer behavior knowledge for effective sports and event marketing 2010 New York: Routledge 59 85

[B20] Witty K White A Tackling men's health: implementation of a male health service in a rugby stadium setting Comm Pract 2011 10 29 32

[B21] Pringle A Zwolinsky S Smith A Robertson S McKenna J White A The pre-adoption demographic and health profiles of men participating in a programme of men's health delivered in English Premier League football clubs Public Health 2011 10 411 416 10.1016/j.puhe.2011.04.013 21726882

[B22] Brady AJB Perry C Murdoch DL McKay G Sustained benefits of a health project for middle-aged football supporters at Glasgow Celtic and Glasgow Rangers Football Clubs Eur Heart J 2010 10 2966 2968 21192349

[B23] Priest N Armstrong R Doyle J Waters E Policy interventions implemented through sporting organisations for promoting healthy behaviour change Cochrane Database Syst Rev 2008 10 doi: 10.1002/14651858.CD004809.pub3. Art. No. CD004809 10.1002/14651858.CD004809.pub3 PMC6464902 18646111

[B24] Priest N Armstrong R Doyle J Waters E Interventions implemented through sporting organisations for increasing participation in sport Cochrane Database Syst Rev 2008 10 doi:10.1002/14651858.CD004812.pub3. Art. No. CD004812 10.1002/14651858.CD004812.pub3 PMC13273799 18646112

[B25] Young MD Morgan PJ Plotnikoff RC Callister R Collins CE Effectiveness of male-only weight loss and weight loss maintenance interventions: a systematic review with meta-analysis Obes Rev 2012 10 393 408 10.1111/j.1467-789X.2011.00967.x 22212529

[B26] Pringle AF Zwolinsky S McKenna J Daly-Smith A Robertson S White A Effect of a national programme of men's health delivered in English Premier League football clubs Public Health 2013 10 18 26 10.1016/j.puhe.2012.10.012 23219262

[B27] Zwolinsky S Pringle A Daly-Smith A McKenna J Robertson S White A Associations between daily sitting time and the combinations of lifestyle risk factors in men J Men’s Health 2012 10 261 267 10.1016/j.jomh.2012.02.003

[B28] Zwolinsky S McKenna J Pringle A Daly-Smith A Robertson S White A Optimizing lifestyles for men regarded as "hard-to-reach" through top-flight football/soccer clubs Health Ed Res 2013 10 405 413 10.1093/her/cys108 23193195

[B29] Craig P Dieppe P Macintyre S Mitchie S Nazareth I Petticrew M Developing and evaluating complex interventions: the new Medical Research Council guidance Brit Med J 2008 10 doi:10.1136/bmj.a1655 10.1136/bmj.a1655 PMC2769032 18824488

[B30] Wyke S Hunt K Gray CM The FFIT team Football Fans in Training (FFIT): a pragmatic randomised controlled trial of a gender-sensitised weight loss and healthy living programme delivered to men aged 35–65 years by Scottish Premier League football clubs Lancet 2012 http://www.thelancet.com/protocol-reviews/11PRT8506 10.1016/S0140-6736(13)62420-4 PMC4524002 24457205

[B31] Calasanti T Pietila I Ojala H King N Men, bodily control and health behaviors: the importance of age Health Psychol 2013 10 15 23 23316849 10.1037/a0029300

[B32] Gray CM Anderson AS Clarke AM Dalziel A Hunt K Leishman J Addressing male obesity: an evaluation of a group-based weight management intervention for Scottish men J Men's Health 2009 10 70 81 10.1016/j.jomh.2008.11.002

[B33] Gray CM Hunt K Lorimer K Anderson AS Benzeval M Wyke S Words matter: a qualitative investigation of which weight status terms are acceptable and motivate weight loss when used by health professionals BMC Public Health 2011 10 1 9 10.1186/1471-2458-11-1 21714892 PMC3142235

[B34] Department of Health Start Active, Stay Active: a report on physical activity for health from the four home countries' Chief Medical Officers 2011 London: Department of Health

[B35] Thomas S Reading J Shephard RJ Revision of the physical activity readiness questionnaire (PAR-Q) Can J Sport Sci 1992 10 338 345 1330274

[B36] Fitzsimons CF Baker G Gray SR Nimmo MA Mutrie N Does physical activity counselling enhance the effects of a pedometer-based intervention over the long-term: 12-month findings from the Walking for Wellbeing in the West study BMC Public Health 2012 10 doi:10.1186/1471-2458-12-206 10.1186/1471-2458-12-206 PMC3349531 22429600

[B37] National Institute for Health and Clinical Excellence Obesity: the prevention, identification, assessment and management of overweight and obesity in adults and children 2006 NICE http://guidance.nice.org.uk/CG43/guidance 22497033

[B38] Scottish Intercollegiate Guidelines Network Management of obesity: a national clinical guideline 2010 Edinburgh: Scottish Intercollegiate Guidelines Network

[B39] Michie S Abraham C Whittington C McAteer J Gupta S Effective techniques in healthy eating and physical activity interventions: a meta-regression Health Psychol 2009 10 690 701 19916637 10.1037/a0016136

[B40] Greaves CJ Sheppard KE Abraham C Hardeman W Roden M Evans PH Systematic review of reviews of intervention components associated with increased effectiveness in dietary and physical activity interventions BMC Public Health 2011 10 doi:10.1186/1471-2458-11-119 10.1186/1471-2458-11-119 PMC3048531 21333011

[B41] Chapple A Ziebland S The role of humor for men with testicular cancer Qual Health Res 2004 10 1123 1139 10.1177/1049732304267455 15359047

[B42] Williams R 'Having a laugh': masculinities, health and humour Nurs Inq 2009 10 74 81 10.1111/j.1440-1800.2009.00437.x 19228306

[B43] Morgan PJ Warren JM Lubans DR Collins CE Callister R Engaging men in weight loss: experiences of men who participated in the male only SHED-IT pilot study Obesity Res Clin Pract 2011 10 e239 e248 10.1016/j.orcp.2011.03.002 24331106

[B44] British Heart Foundation So you want to lose weight… for good 2009 London: British Heart Foundation

[B45] Craig CL Marshall AL Sjostrom M Bauman AE Booth ML Ainsworth BE International physical activity questionnaire: 12-country reliability and validity Med Sci Sports Exerc 2003 10 1381 1395 10.1249/01.MSS.0000078924.61453.FB 12900694

[B46] Roe L Strong C Whiteside C Neil A Mant D Dietary intervention in primary care: validity of the DINE method for diet assessment Fam Pract 1994 10 375 381 10.1093/fampra/11.4.375 7895964

[B47] Emslie C Lewars H Batty GD Hunt K Are there gender differences in levels of heavy, binge and problem drinking? Evidence from three generations in the West of Scotland Public Health 2009 10 12 14 10.1016/j.puhe.2008.06.001 19049841 PMC2637302

[B48] Rosenberg M Society and the adolescent self-image 1965 Princeton, NJ: Princeton University Press

[B49] Thompson ER Development and validation of an internationally reliable short form of the positive and negative affect schedule (PANAS) J Cross-Cult Psychol 2007 10 227 242 10.1177/0022022106297301

[B50] Gandek B Ware JE Aaronson NK Apolone G Bjorner JB Brazier JE Cross-validation of item selection and scoring for the SF-12 Health Survey in nine countries: results from the IQOLA Project J Clin Epidemiol 1998 10 1171 1178 10.1016/S0895-4356(98)00109-7 9817135

[B51] Ritchie J Lewis J Qualitative Research Practice 2003 London: Sage

[B52] Bowcott O UK snow: fresh blizzards grip Scotland with hundreds stranded in cars Guardian 2010 http://www.guardian.co.uk/uk/2010/dec/06/scotland-snow-uk-weather

[B53] Bandura A Social cognitive theory of self-regulation Organ Behav Hum Decis Process 1991 10 248 287 10.1016/0749-5978(91)90022-L

[B54] Morgan PJ Lubans DR Callister R Okely AD Burrows TL Fletcher R The 'Healthy Dads, Healthy Kids' randomized controlled trial: efficacy of a healthy lifestyle program for overweight fathers and their children Int J Obes 2011 10 436 447 10.1038/ijo.2010.151 20697417

[B55] Fjeldsoe B Neuhaus M Winkler E Eakin E Systematic review of maintenance of behavior change following physical activity and dietary interventions Health Psychol 2011 10 99 109 21299298 10.1037/a0021974

[B56] Peterson JC Pirraglia PA Wells MT Charlson ME Attrition in longitudinal randomized controlled trials: home visits make a difference BMC Med Res Methodol 2012 10 doi:10.1186/1471-2288-12-178 10.1186/1471-2288-12-178 PMC3536670 23176384

[B57] Morgan PJ Lubans DR Collins CE Warren JM Callister R The SHED-IT randomized controlled trial: evaluation of an Internet-based weight-loss program for men Obesity 2009 10 2025 2032 10.1038/oby.2009.85 19343018

[B58] Leslie WS Lean ME Baillie HM Hankey CR Weight management: a comparison of existing dietary approaches in a work-site setting Int J Obes 2002 10 1469 1475 10.1038/sj.ijo.0802153 12439649

[B59] Pritchard JE Nowson CA Wark JD A worksite program for overweight middle-aged men achieves lesser weight loss with exercise than with dietary change J Am Diet Assoc 1997 10 37 42 10.1016/S0002-8223(97)00015-1 8990415

[B60] Morgan PJ Lubans DR Collins CE Warren JM Callister R 12-month outcomes and process evaluation of the SHED-IT RCT: an internet-based weight loss program targeting men Obesity 2011 10 142 151 10.1038/oby.2010.119 20523304

[B61] Frey-Hewitt B Vranizan KM Dreon DM Wood PD The effect of weight loss by dieting or exercise on resting metabolic rate in overweight men Int J Obes 1990 10 327 334 2361810

[B62] Glasgow RE Klesges LM Dzewaltowski DA Estabrooks PA Vogt TM Evaluating the impact of health promotion programs: using the RE-AIM framework to form summary measures for decision making involving complex issues Health Ed Res 2006 10 688 694 10.1093/her/cyl081 16945984

[B63] Shephard RJ Limits to the measurement of habitual physical activity by questionnaires Brit J Sports Med 2003 10 197 206 10.1136/bjsm.37.3.197 12782543 PMC1724653

[B64] Torgerson DJ Contamination in trials: is cluster randomisation the answer? Br Med J 2001 10 355 357 10.1136/bmj.322.7282.355 11159665 PMC1119583

